# Nutritional Status of Patients with Tuberculosis and Associated Factors in the Health Centre Region of Burkina Faso

**DOI:** 10.3390/nu12092540

**Published:** 2020-08-21

**Authors:** Beatrice B. Musuenge, Ghislain G. Poda, Pei-Chun Chen

**Affiliations:** 1 Department of Public Health, China Medical University, 91 Hsueh-Shih Road, North District, Taichung City 40402, Taiwan; bmusuenge@yahoo.fr; 2 Public Health Department, University of Ouagadougou, Ouagadougou 03 BP 7021, Burkina Faso; podaghis@yahoo.fr

**Keywords:** tuberculosis, undernutrition, adults, associated factors, Burkina Faso

## Abstract

Extreme hunger and poverty remain a significant barrier to maintaining a normal and healthy life, and increase the burden of tuberculosis (TB) in sub-Saharan African countries. The purpose of this study was to assess the prevalence and factors associated with undernutrition among adult patients with TB in Burkina Faso. In the Health Centre Region of Burkina Faso, we conducted a cross-sectional survey with face-to-face interviews of patients aged 15 years and older with TB (*n* = 302), from March to April 2019. Undernutrition was defined as a body mass index of <18.5 kg/m^2^. Multiple logistic regression analyses were performed to determine the correlates of undernutrition. The prevalence of undernutrition was 35.8%, in which severe, moderate, and mild thinness were 7.7%, 8.9%, and 19.5%, respectively. Low food frequency per day (adjusted odds ratio [aOR] = 3.28, confidence interval [CI]: 1.42–7.55, *p* = 0.005), human immunodeficiency virus infection (aOR = 6.18, CI: 2.26–16.88, *p* < 0.001), and diabetes mellitus (aOR = 17.52, 95% CI: 1.88–162.97, *p* = 0.012) and young age (45–54 years vs. 65 years and older, aOR = 2.93, 95% CI: 1.11–7.70, *p* = 0.029) were associated with increased odds of undernutrition. We concluded that the assessment of comorbidity and nutritional status should be part of the treatment for adult TB patients.

## 1. Introduction

Despite scientific progress and the availability of drugs, tuberculosis (TB) remains a great challenge and a global public health concern. According to the World Health Organization (WHO) report, there were over 10 million new cases of active TB each year, and 1.5 million died from the disease worldwide in 2018 [1]. More than 80% of patients with TB live in low-and middle-income countries, particularly Asia and sub-Saharan Africa countries, where poverty and extreme hunger are endemic and devastating public health issue [2], including Burkina Faso [3].

Patients with active TB are more likely to be emaciated [4] and have a low body mass index (BMI), with a value less than 18.5 kg/m^2^ being considered as an index of undernutrition [5]. TB is an infectious disease caused by bacillus mycobacterium tuberculosis, which mostly occurs among the poorest populations. Patients with TB are more likely to have a reduction in appetite, nutrient malabsorption, micronutrient malabsorption, and impaired metabolism, which could lead to emaciation. Besides, weight loss is associated with illness and gastrointestinal disorders such as anorexia, nausea and vomiting, TB, and human immunodeficiency virus (HIV) infection. Metabolism impairment leading to weight loss and the exacerbated poor nutritional status has been reported previously [4,6,7,8]. An Indian study reported that patients with TB were 11-fold more likely to have a BMI of <18.5 kg/m^2^ than the control group [9]. Globally, the WHO estimated that approximately one-quarter of all new TB cases were attributable to undernutrition in 2018; in contrast, individuals with undernutrition are at three times increased risk of contracting active TB [1]. These observations indicate that undernutrition among patients infected with TB remains a serious and devastating issue.

It is well known that TB and malnutrition are interconnected in a complex bidirectional relationship, which makes both of these conditions worse [6,10,11]. Malnutrition weakens the immune system, and increases the risk of progression from latent TB infection to active TB; TB also predisposes and worsens malnutrition [12,13,14,15]. Undernutrition is a risk factor for the increased severity of TB [4,16] and unfavorable treatment outcomes, including TB delayed recovery/TB relapse and mortality [17,18,19,20,21]. Previous studies revealed that TB patients who were treated and received adequate dietary intake and supplementary nutritional care experienced an earlier significant nutritional change of BMI restoration, which is associated with evident immune system improvement and treatment success outcomes [4,16,17,22,23,24,25,26].

Previous studies have underlined the increasing prevalence of undernutrition in patients with TB; however, this prevalence varied widely among continents, countries, and regions [6,13,20,26,27], with an estimated prevalence ranging from 50% to 87.0%. In Burkina Faso, most of the previous studies evaluated malnutrition in children under five [28,29] and children with HIV [30]. To the best of our knowledge, no studies have reported this issue in adults with TB.

Combatting hunger, undernutrition, and TB is among the 8 United Nations Sustainable Development Goals (UN-SDGs) by 2030. Therefore, an understanding of nutritional status in adults and its correlates could provide evidence to support the development of strategies for TB management. The objective of this study was to assess the prevalence and associated factors of undernutrition in adults with TB, which is a current challenge in Burkina Faso.

## 2. Materials and Methods

### 2.1. Study Design and Setting

A quantitative cross-sectional study was carried out in Ouagadougou, the capital city of Burkina Faso. According to the report of the United Nations, the total population in Burkina Faso was estimated at 20.9 million people, of which 2.2 million live in the capital city as of 2020 [31]. The data were collected in the Health Centre Region, where patients with TB were identified by screening in the national TB diagnosis and treatment centre.

### 2.2. Selection of Study Subjects

The study included patients aged 15 years or older who were newly diagnosed with active pulmonary TB by sputum microscopy, and received anti-TB treatment under a directly observed treatment short course (DOTS), at the five selected district health facilities in Ouagadougou. The use of 15 years of age rather than 18 years as the cut-off to define adult patients in this study followed the WHO definitions for TB surveillance and management [32]. In TB diagnosis and treatment, the disease in individuals aged less than 0–14 years (also called paediatric tuberculosis) was considered differently from that in older children and adults. Subjects were excluded if they (1) were not able to read and speak French, (2) had extra-pulmonary TB, (3) were in the continuation phase of TB treatment, (4) were pregnant or breast-feeding, or (5) had relapsed. The sample size was calculated using Epi-info software version 7, assuming a 95% confidence level, 50% of expected frequency, and a design margin error of 5%. The required sample size was 290 patients, but 302 patients met the inclusion criteria. We collected more than the expected number because numerous TB patients were willing to participate in the study.

### 2.3. Data Collection

A face-to-face interview conducted by four well-trained clinical nurses was used to collect patient information. We used an adapted questionnaire from previous studies’ findings [12,27,33] to meet the study goal. The initial questionnaire was prepared in English. It was then translated into French, the official language of Burkina Faso, and back translated by one of the co-authors and his colleagues from university who speak fluent English and French. The questionnaire consisted of four parts: (i) the socio-demographic characteristics of the patients, (ii) the lifestyle of tuberculosis patients, (iii) the health status of adult tuberculosis patients, and (iv) the nutritional status of adult tuberculosis patients.

The socio-demographic information included age, sex (male and female subjects), marital status (married and single, including divorced/separated and widowed), educational level (no education, primary or secondary school, and university), occupational status (employed, self-employed and unemployed), monthly income (<200,000, 101,000–200,000, and >201,000 FCFA), family size (2–4, 5–8, and >9 people, and residence (living in rural or urban areas). Family size was defined as the number of people living in the same house. The lifestyle information of TB patients included alcohol consumption and cigarette smoking (never, former and current). A family history of TB was defined as living with anyone with TB (yes or no). The health status (or comorbidity) of patients was determined by the duration of anti-TB treatment (<4 or ≥4 weeks). An eating problem was defined as the presence of an illness like nausea, vomiting, appetite loss, abdominal pain, or dysphagia (yes or no). Anxiety/sadness was defined as feeling stressed (yes or no). Information on comorbidities was also collected, including HIV infection, diabetes mellitus, hypertension, and hepatitis B infection (yes or no). The dietary information of patients was evaluated after a 24-h recall before the survey for what they had eaten. TB patients’ food intake was assessed by the frequency of meal consumption (1–2 meals or more than two meals per day), meal or food diversity, meals reduced (amount of food intake per day), and receiving dietary care or counselling (yes or no).

Weight was measured in kilograms to the nearest 0.1 kg using a digital standing scale, according to the standard examination procedures, which consists of patients standing in the centre of the scale platform facing the recorders, looking straight ahead, and placing their hands at their side. Height, which is a maximum vertical size, was measured in centimetres to the nearest 0.1 cm using the stature meter (Microtoise), and was recorded using the standard examination procedures. Patients’ shoes were removed to ensure the accuracy of the measures. Patients were positioned with their feet together and flat on the ground, arms loosely at their side, legs straight, and shoulder blades, buttocks, and heels touching the backboard [34].

Three experts conducted a content validly test in Burkina Faso, and the index for this questionnaire was 0.9, indicating that the tools were clear and relevant. Subsequently, a pilot study was conducted among 30 patients with TB. The purpose of this pilot study was to assess feasibility and time, and to improve upon the study design before the performance of a full-scale research project. Patients’ data were kept anonymous and in confidentiality, to ensure the privacy of the study respondents at every step.

### 2.4. Outcome Variables

The outcome variable of this study was the nutritional status defined by BMI. Height and body weight were used to calculate BMI (kg/m^2^). Patients with a BMI < 18.5 kg/m^2^ were defined as having undernutrition. Undernutrition is a poor nutritional status defined by having a body mass index (BMI) lower than 18.5 kg/m^2^ due to imbalanced food intake and an abnormal utilization of nutrients, referring to all deviations from adequate nutrition, including wasting away, stunting, or the deficiency of micronutrients (severe, moderate and mild thinness) [35]. According to WHO, severe, moderate, and mild thinness are defined as a BMI < 16.0 kg/m^2^, 16.0–16.99 kg/m^2^, and 17.0–18.49 kg/m^2^, respectively [5].

### 2.5. Ethical Considerations

Ethical clearance was obtained from, and approved by, the Institutional Review Board of Burkina Faso, National Health Research Ethics Committees (Reference number: 2019-02-020/MS/SG/DGS/DN) and the head of the Health Centre Region in Burkina Faso 2019 (Reference number: 2019-034/MS/RCEN/DRSC). Moreover, administrative authorization was obtained from the Health Centre Region in Ouagadougou. Written informed consent was obtained from the patients before the data collection.

### 2.6. Statistical Analysis

The investigators first checked the data after entering them in Microsoft Excel 2013 (Microsoft Corporation Inc. USA) for codification. The data were then exported for further analysis in SPSS (Statistical Package for the Social Sciences) software, version 23.0. Descriptive statistical analysis was performed to estimate the mean and standard deviation for continuous data, and the percentage frequency for categorical data. Differences in characteristics in the categorical scale between participants, with or without undernutrition, were examined using the Pearson Chi-square (χ^2^) test. Logistic regression analyses, which produced the odds ratios (ORs) and 95% confidence intervals (CIs), were performed, to assess the association between each of the potential correlates and undernutrition. Unadjusted logistic regression analysis was performed with all variables listed in the data collection. We classified age into six categories, 15–24, 25–34, 35–44, 45–54, 55–64, and 65 and older, which is consistent with the age groups applied in the WHO report of TB surveillance data [32]. To evaluate the correlates of undernutrition, we performed multiple logistic regression models, in which variables statistically significant, associated with undernutrition in the simple logistic regression analysis, were included. The statistically significant level was set at a two-sided *p* value < 0.05.

## 3. Results

### 3.1. Nutritional Status of Adult TB Patients

The overall prevalence of undernutrition among TB patients was 35.8% (BMI < 18.5 kg/m^2^) (Table 1). Overall, 7.3 percent of patients had severe thinness (BMI < 16.0 kg/m^2^), 8.9% moderate thinness (16.0–16.99 kg/m^2^), and 19.5% mild thinness (17.0–18.49 kg/m^2^). The findings also showed that 2.6% were overweight (BMI > 25 kg/m^2^), and 61.6% were of normal weight (BMI 18.5–24.9 kg/m^2^). The distribution of BMI was not significantly different between male and female patients (*p* = 0.78).

### 3.2. Socio-Demographic Characteristics and Lifestyle Variables

The majority (65.6%) of the study subjects were male patients (Table 2). The mean age of the patients was 43.6 (standard deviation, 15.3) years (minimum = 15 years; maximum = 89 years). Of all patients, 66.9% were educated, 82.5% had a monthly income of ≤100,000 West African CFA (approximately USD 172), 83.8% resided in an urban area, and 78.5% were married (Table 2). More than half of the patients had an education level of primary/secondary school, whereas only 3.6% of patients have received university education (Table 2). More than half of the patients (58.6%) were self-employed, and 25.8% were unemployed. Fifty-six-point three percent of patients had a family size of 5–8 people, and 40.4% had a family size of 1–4 people. The prevalence of current alcohol consumption (57.9%) and cigarette smoking (57.0%) was high. The frequency distribution of all the variables was not statistically significantly different between patients with and without undernutrition (Table 2).

### 3.3. Nutritional Intervention

Most of the patients (97.4%) had received dietary counselling, 62.3% had nutritional care and support, and 88.7% had meal diversity (Table 3). However, most patients did not have a high enough daily food intake: 84.1% consumed only 1 or 2 meals per day, and 86.4% experienced a reduction in the amount of food intake (Table 3). Relative to subjects without undernutrition, those with undernutrition tended to have a lower food frequency per day (1 or 2 meals per day, 90.7% vs. 80.4%, *p* = 0.021), and their meals reduced (91.7% vs. 83.5%, *p* = 0.054).

### 3.4. Health Condition of Adult TB Patients

Almost all respondents (99%) were treated in outpatient clinics, and 93.0% received anti-TB treatment for more than four weeks during the DOTS period (Table 3). Nearly 57.0% were anxious, and 48.0% had a history of TB in the family. Of all patients, only 4.3% had eating problems. The prevalence of HIV infection was 7.6%, and the prevalence of hypertension and diabetes was both 2%. The prevalence of hepatitis B infection was 1%. Relative to patients with normal weight, those with undernutrition had a much higher prevalence of HIV infection (*p* < 0.001) and diabetes (*p* = 0.023).

### 3.5. Odds Ratios for the Factors Associated with Undernutrition

In the unadjusted logistic regression model, adult patients aged 45–54 years had a borderline significantly elevated odds of undernutrition, as compared with those aged 65 years and above (OR = 2.46, 95% CI: 1.00–6.06, *p* = 0.051) (Table 4). In addition, less daily food frequency intake (OR = 2.39, 95% CI: 1.14–5.01, *p* = 0.021), HIV infection (OR = 5.85, 95% CI: 2.23–15.34, *p* < 0.001), and diabetes (OR = 9.37, 95% CI: 1.08–81.26, *p* = 0.042) were associated with increased odds of undernutrition. Patients with reduced meals had a borderline significant increase in odds of having undernutrition (OR = 2.17, 95% CI: 0.99–4.74, *p* = 0.051). However, current smokers tended to have decreased odds of having undernutrition, but the association was not statistically significant (OR = 0.43, 95% CI: 0.18–1.01, *p* = 0.052), as compared to those who had never smoked. We did not observe significant associations between other factors and the odds of having undernutrition. The ORs for the groups of no education and elementary/secondary school were both approximately 1.50. They did not reach statistical significance, indicating a non-association between education levels and undernutrition.

Table 5 shows the results of the multiple logistic regression analysis. In the results of a simple logistic regression model shown in Table 4, age, daily food frequency, HIV infection, and diabetes mellitus were significantly associated with undernutrition. These variables were included in the multiple logistic regression model. After adjusting for these variables, the adjusted OR (aOR) of undernutrition in subjects aged 45–54 years was 2.93 (95% CI: 1.11–7.70, *p* = 0.029), as compared with those aged 65 years and older. The aOR for the low frequency of food intake (1–2 meals per day), compared to adequate food intake frequency (more than two meals per day), was 3.28 (95% CI: 1.42–7.55, *p* = 0.005). HIV infection was associated with greater odds of undernutrition (aOR = 6.18, 95% CI: 2.26–16.88, *p* < 0.001). Patients with diabetes had increased odds of undernutrition, as compared with those without diabetes (aOR = 17.52, 95% CI: 1.88–162.97, *p* = 0.012).

## 4. Discussion

This study showed that, in TB patients undergoing anti-TB treatment in Ouagadougou, Burkina Faso, 35.8% suffered from undernutrition, and 2.6% were overweight. Our findings highlighted that HIV infection, diabetes mellitus, and lower daily food frequency were independently associated with undernutrition in adults with TB. The prevalence of undernutrition that we observed in this study was higher than that of the general population (10%) of Burkina Faso [36].

Previous studies have also reported a high prevalence of undernutrition among adult TB populations in other countries. Several studies conducted in Sub-Saharan African countries revealed that 46.0% in Uganda [10], 43% in Kenya [37], of TB patients were underweight. In Malawi, 57% of adult TB patients on admission [20] were undernourished, and in Ghana, 51% [27] were undernourished at the time that treatment was initiated. A cross-sectional study conducted in Ethiopia reported that 57.17% of adult TB patients were undernourished [38]. A high prevalence of undernutrition among TB patients has also been reported in Asia Pacific countries—66% in India [6], and 87% in Indonesia [13]. A study showed that all patients newly diagnosed with TB in Brazil were undernourished at baseline [39].

However, a direct comparison of the prevalence of malnutrition among studies may be inappropriate, because of the differences in survey methods and inclusion criteria of study subjects. The discrepancy in the prevalence rates reported among studies may be due to differences in socioeconomic and demographic factors, lifestyle, the severity of the disease, and the time when the studies were carried out. Most investigations, including ours, were not nationally representative, and several of the previous studies were based on data from a single hospital. Despite the discrepancy in the estimated prevalence rates among the studies, all studies revealed a high prevalence among TB patients, indicating that undernutrition is a great public health concern, particularly in countries with a high prevalence of TB. The high prevalence of undernutrition reported in the present study may reflect the low socioeconomic and demographic background of TB patients, as displayed in Table 2 and Table 3.

This study found that the majority of adult TB patients consumed a low frequency, 1 to 2 meals per day, and low daily food frequency was associated with increased odds of undernutrition. This finding was consistent with previous studies from Uganda [10], Nepal [10], and Kenya [21], which revealed that adult TB patients did not consume adequate dietary intakes. A potential reason for poor food frequency per day is likely due to the loss of appetite caused by the disease [7,21], which is associated with consumption and weight loss [40]. Several previous studies from India [8] and Gondar (Ethiopia) [41] and Kenya [21] reported that the micronutrients status (including low concentrations of hemoglobin, serum albumin, serum retinol, and serum zinc) of adult TB patients was significantly lower and more pronounced among malnourished TB patients than healthy patients [41]. Thus, it may indicate that undernutrition in TB patients is associated with food intake and nutrient absorption, which both have negative implications on nutritional status and suggests that patients with TB require greater nutritional support than those without TB.

Our observation was consistent with previous studies in Uganda (Mulago) [10] and Ethiopia [38,42], which showed that patients with HIV infection had greater odds of having undernutrition. A possible explanation is that HIV infected patients have a poor appetite. Several studies conducted in other settings reported that TB and HIV infection both allow the sufferer to waste away, and worsen nutritional status [16,43,44]. Besides, patients with both TB and HIV tended to have clinical symptoms related to gastrointestinal disorders and weight loss [16,44]. Low micronutrient concentrations of trace elements related to the nutritional status have been reported in adult patients with TB [8,15,25,43,45], and in those co-infected with HIV [43,46,47,48]. Moreover, strong interaction, due to a combination of HIV, TB, and undernutrition, constitutes a triple problem [44], thereby leading to worsening of pre-existing undernutrition [43,47] and immune system vulnerability.

The findings of the present study showed that diabetes was independently associated with undernutrition in patients with TB. Several studies have shown that TB may cause glucose tolerance impairment [49,50,51,52], metabolic changes, and muscle wasting [51,53]. Furthermore, TB patients with diabetes have worse clinical presentations and more symptoms at onset [50], especially weight loss [53], when compared to TB patients without diabetes. In countries with low and middle incomes, undernutrition among TB patients is associated with increased morbidity and mortality of diabetes. A study conducted in India reported a high prevalence of diabetes among active TB patients at all levels of BMI [54], and more than half (55%) of diabetic patients were ignorant of their diabetic status at the time of TB diagnosis [55]. These observations and our findings suggest that aroutine bidirectional screening for detecting pre-diabetes and HIV infection and assessment of the nutritional status of TB patients are necessary. Early detection of these coexisted health conditions may help to decrease the burden of TB and provide effective nutritional management, as suggested by previous studies [55,56].

In this study, men accounted for 65.6% of patients with TB, representing a male to female ratio of 1.9. There was a high proportion of men in both undernutrition and normal-weight groups (sex ratio, 1.6 and 2.1, respectively). The sex distribution in our sample of TB patients was consistent with that observed in Burkina Faso and globally. The WHO report in 2019 indicated that, globally, the male to female ratio was 2:1 among patients with TB [57]. In the TB case notifications data of Burkina Faso, male patients accounted for 70% of totally new and relapse cases [57]. Potential explanations for the sex differences include sociocultural and biological aspects. Evidence has shown that women, particularly in developing countries, were less likely to report illness and access to TB care and diagnosis [58]. The potential reasons include a lack of resources and support, stigma, and selection bias of healthcare providers due to differences in the perception and report of symptoms among women [58]. Besides, previous studies have suggested that men were more likely to develop the disease after infection with *M. tuberculosis* when getting older, and men tended to report risk factors and unhealthy behaviours associated with the development and progression of TB [59,60,61].

The strength of this study is that the study subjects consisted of patients from the Health Centre Region, rather than a single hospital. Furthermore, many factors were considered as the potential correlates of undernutrition, such as socio-demographic characteristics, nutrition information, and health status. However, this study also has several limitations. First, almost all of our study subjects received TB treatments for more than four weeks during the study period. Thus, it might have resulted in an underestimation of the prevalence of undernutrition among TB patients, because nutritional status commonly improves over time. Second, information regarding health conditions was collected by self-reported questionnaires, which may not be accurate for the items measuring subjective feelings, such as anxiety. Third, this study may not be nationally representative, because of the inclusion of participants from only the Health Centre Region located in the capital city. Indeed, the proportion of urban dwellers was much higher in the present study than that in the entire country (83.8% vs. 30.0% in 2019) [62]. Our observation was consistent with a cross-sectional study of patients with HIV in the Centre region of Burkina Faso in 2013, which also showed a high percentage (66.7%) of study subjects residing in urban areas [63]. An even higher percentage observed in our survey may reflect the rapid increase in the urban population in Burkina Faso over the past few years [62]. Fourth, a previous study has shown that, among current smokers, older people tended to have a longer duration of smoking [64]. We did not collect information on the length of smoking and alcohol drinking, which may have confounded the observed association between age and undernutrition, if these factors were associated with undernutrition. However, the association of the length and intensity of smoking and drinking with undernutrition among adult patients with TB has not been well documented yet. More studies are needed to clarify this issue. Fifth, to avoid misunderstanding and to increase the survey validity, we only included people who read and spoke French, which is the official language of Burkina Faso. Moreover, the data collectors did not speak all of the local languages. The results of this study may, therefore, not be generalizable to people who do not speak French.

## 5. Conclusions

The findings of our study showed that in Ouagadougou, Burkina Faso, newly diagnosed adult patients with TB had poor nutritional status. Despite the implementation of counselling and nutritional care among TB patients in Burkina Faso, a high proportion of patients had a low frequency of food intake, and the nutrition care and counselling service did not improve the nutrition status. These observations suggest that public health actions are needed to improve the quality of services, and strengthen nutritional programs among adult TB patients. These may include the integration of food assistance in the TB National Program Strategic Plan; advocacy to Partners and Ministry of Health to mobilize resources for food assistance; the development of guidelines for nutrition care and support to improve the quality of nutrition care; providing health education to improve awareness of undernutrition and its consequences for patients with TB and their family members. Moreover, we observed a strong association between HIV infection and diabetes, with undernutrition in patients with TB. These findings suggest that the regular assessment of nutritional status and early screening and control for comorbidities, particularly HIV and diabetes, should be regarded as crucial components of TB treatment and management, as recommended by the World Health Organization.

## Figures and Tables

**Table 1:** Nutritional status in male and female TB patients undergoing anti-TB treatment in Ouagadougou, Burkina Faso.

Characteristics	BMI (kg/m^2^)		
	Female Patients	Male Patients	Total
	*n* (%)	*n* (%)	*n* (%)
Undernutrition	41 (39.4)	67 (33.8)	108 (35.8)
Severe (BMI < 16.0 kg/m^2^)	8 (7.7)	14 (7.1)	22 (7.3)
Moderate (BMI = 16.0–16.99 kg/m^2^)	8 (7.7)	19 (9.6)	27 (8.9)
Mild (BMI = 17.0–18.49 kg/m^2^)	24 (23.1)	35 (17.7)	59 (19.5)
Normal weight (BMI = 18.5–24.99 kg/m^2^)	62 (59.6)	124 (62.6)	186 (61.6)
Overweight (BMI = 25.0–29.99 kg/m^2^)	2 (1.9)	6 (3.0)	8 (2.6)

BMI indicates body mass index; TB, tuberculosis.

**Table 2:** Socio-demographics and lifestyle variables by nutritional status in adult TB patients undergoing anti-TB treatment in Ouagadougou, Burkina Faso.

Variables	Categories	Under Weight (BMI < 18.5 kg/m^2^)	Normal Weight (BMI ≥ 18.5 kg/m^2^)	Total *n* (%)	*p*-Value
		*n* (%)	*n* (%)		
Age (years)	Mean = 43.6; Standard deviation (SD) = ±15.3; Maximum = 89 years				
	15–24	12 (11.1)	15 (7.7)	27 (8.9)	0.117
	25–34	20 (18.5)	40 (20.6)	60 (19.9)	
	35–44	31 (28.7)	58 (29.9)	89 (29.5)	
	45–54	31 (28.7)	35 (18.0)	66 (21.9)	
	55–64	5 (4.6)	21 (10.8)	26 (8.6)	
	65 and more	9 (8.3)	25 (12.9)	34 (11.3)	
Sex	Female subjects	41 (38.0)	63 (32.5)	104 (34.4)	0.377
	Male subjects	67 (62.0)	131 (67.0)	198 (65.6)	
Education	No education	36 (33.3)	64 (33.0)	100 (33.1)	0.836
	Primary and/or secondary school	69 (63.9)	122 (62.9)	191 (63.2)	
	University	3 (2.8)	8 (4.1)	11 (3.6)	
Occupational status	Employed	12 (11.1)	35 (18.0)	47 (15.6)	0.277
	Self employed	66 (61.1)	111 (57.2)	177 (58.6)	
	Unemployed	30 (27.8)	48 (24.7)	78 (25.8)	
Monthly income (FCFA) *	≤100,000	91 (84.3)	158 (81.4)	249 (82.5)	0.377
	101,000–200,000	16 (14.8)	29 (14.9)	45 (14.9)	
	≥201,000	1 (0.9)	7 (3.6)	8 (2.6)	
Family size	1–4	47 (43.5)	75 (38.7)	122 (40.4)	0.687
	5–8	58 (53.7)	112 (57.7)	170 (56.3)	
	≥9	3 (2.8)	7 (3.6)	10 (3.3)	
Residence area	Urban	88 (81.5)	165 (85.1)	253 (83.8)	0.421
	Rural	20 (18.5)	29 (14.9)	49 (16.2)	
Marital status	Married	87 (80.6)	150 (77.3)	237 (78.5)	0.561
	Unmarried	21 (19.4)	44 (22.7)	65 (21.5)	
Alcohol drinking	Never	11 (10.2)	13 (6.7)	24 (7.9)	0.334
	Former	32 (29.6)	71 (36.6)	103 (34.1)	
	Current	65 (60.2)	110 (56.7)	175 (57.9)	
Cigarette smoking	Never	14 (13.0)	15 (7.7)	29 (9.6)	0.107
	Former	29 (26.9)	72 (37.1)	101 (33.4)	
	Current	65 (60.2)	107 (55.2)	172 (57.0)	

TB indicates tuberculosis; * 1 US dollar = 596.8 West African CFA Francs on 20 March 2020.

**Table 3:** Dietary information and health conditions by nutritional status in adult TB patients undergoing anti-TB treatment in Ouagadougou, Burkina Faso.

Variables	Categories	Under Weight (BMI < 18.5 kg/m^2^)	Normal Weight (BMI ≥ 18.5 kg/m^2^)	Total *n* (%)	*p*-Value
		*n* (%)	*n* (%)		
**Dietary Information**					
Food intake frequency	1–2	98 (90.7)	156 (80.4)	254 (84.1)	0.021
	3–4	10 (9.3)	38 (19.6)	48 (15.9)	
Eating balanced	Yes	100 (92.6)	168 (86.6)	268 (88.7)	0.131
Meals reduced	Yes	99 (91.7)	162 (83.5)	261 (86.4)	0.054
Dietary counselling	Yes	103 (95.4)	191 (98.5)	294 (97.4)	0.140
Nutrition care and support	Yes	66 (61.1)	122 (62.9)	188 (62.3)	0.805
**Health Conditions**					
Duration anti-TB medication	<4 Weeks	11 (10.2)	10 (5.2)	21 (7.0)	0.105
	>4 Weeks	97 (89.8)	184 (94.8)	281 (93.0)	
Family history of TB	Yes	54 (50.0)	91 (46.9)	145 (48.0)	0.632
Eating problem	Yes	7 (6.5)	6 (3.1)	13 (4.3)	0.235
Anxiety	Yes	69 (63.9)	103 (53.1)	172 (57.0)	0.089
HIV infection	Yes	17 (15.7)	6 (3.1)	23 (7.6)	<0.001
Diabetes	Yes	5 (4.6)	1 (0.5)	6 (2.0)	0.023
Hypertension	Yes	4 (3.7)	2 (1.0)	6 (2.0)	0.192
Hepatitis B infection	Yes	2 (1.9)	1 (0.5)	3 (1.0)	0. 292

TB indicates tuberculosis.

**Table 4:** Logistic regression analysis for factors associated with undernutrition in adult TB patients undergoing anti-TB treatment in Ouagadougou, Burkina Faso.

Characteristics	Categories	Crude Odds Ratio	95% Confidence Interval	*p*-Value
Age (years)	15–24 vs. 65 and more	2.22	0.76–6.51	0.146
	25–34 vs. 65 and more	1.39	0.55–3.53	0.490
	35–44 vs. 65 and more	1.49	0.62–3.57	0.378
	45–54 vs. 65 and more	2.46	1.00–6.06	0.051
	55–64 vs. 65 and more	0.66	0.19–2.28	0.513
Sex	Female vs. males	1.27	0.78–2.08	0.336
Education level	No education vs. university	1.50	0.37–6.01	0.567
	Elementary/secondary school vs. university	1.51	0.39–5.87	0.554
Occupation status	Self-employed vs. employed	1.73	0.84–3.57	0.136
	Unemployed vs. employed	1.82	0.82–4.05	0.141
Monthly income (FCFA) *	101,000–200,000 vs. ≤100,000	0.96	0.49–1.86	0.225
	≥201,000 vs. ≤100,000	0.25	0.03–2.05	0.248
Family size	5–8 vs. 1–4	0.82	0.51–1.34	0.439
	≥9 vs. 1–4	0.68	0.17–2.77	0.595
Residence area	Rural vs. urban	1.29	0.69–2.42	0.421
Marital status	Unmarried vs. married	0.82	0.46–1.47	0.512
Alcohol drinking	Former vs. never	0.53	0.21–1.32	0.172
	Current vs. never	0.70	0.29–1.65	0.413
Cigarette smoking	Former vs. never	0.65	0.29–1.43	0.287
	Current vs. never	0.43	0.18–1.01	0.052
Daily food frequency	1–2 vs. 3–4	2.39	1.14–5.01	0.021
Eating balanced	Yes vs. no	1.93	0.84–4.44	0.119
Meals reduced	Yes vs. no	2.17	0.99–4.74	0.051
Dietary counselling	Yes vs. no	0.32	0.07–1.38	0.128
Nutrition care	Yes vs. no	0.93	0.57–1.50	0.760
Duration of anti TB treatment	<4 Weeks vs. ≥4 weeks	2.09	0.85–5.08	0.106
Family history of TB	Yes vs. no	1.13	0.71–1.81	0.606
Eating problem	Yes vs. no	0.46	0.15–1.41	0.174
HIV infection	Yes vs. no	5.85	2.23–15.34	<0.001
Diabetes mellitus	Yes vs. no	9.37	1.08–81.26	0.042
Hypertension	Yes vs. no	0.27	0.05–1.50	0.135
Hepatitis B infection	Yes vs. no	0.27	0.02–3.06	0.294
Anxiety	Yes vs. no	1.56	0.96–2.53	0.070

HIV indicates human immunodeficiency virus; TB, tuberculosis; * 1 US dollar = 596.8 West African CFA Francs on 20 March 2020.

**Table 5:** Multiple logistic regression analysis for factors associated with undernutrition in adult TB patients undergoing anti-TB treatment in Ouagadougou, Burkina Faso.

Characteristics	Categories	Adjusted Odds Ratio *	95% Confidence Interval	*p*-Value
Age (years)	15–24 vs. 65 and more	3.02	0.96–9.52	0.059
	25–34 vs. 65 and more	1.46	0.54–3.92	0.457
	35–44 vs. 65 and more	1.36	0.53–3.53	0.525
	45–54 vs. 65 and more	2.93	1.11–7.70	0.029
	55–64 vs. 65 and more	0.88	0.24–3.16	0.841
Daily food frequency	1–2 vs. 3–4	3.28	1.42–7.55	0.005
HIV infection	Yes vs. no	6.18	2.26–16.88	<0.001
Diabetes mellitus	Yes vs. no	17.52	1.88–162.97	0.012

HIV indicates human immunodeficiency virus; TB, tuberculosis; * The model was mutually adjusted for all variables in the table.
